# Toward healthy and sustainable diets for the 21st century: Importance of sociocultural and economic considerations

**DOI:** 10.1073/pnas.2219272120

**Published:** 2023-06-12

**Authors:** Sander Biesbroek, Frans J. Kok, Adele R. Tufford, Martin W. Bloem, Nicole Darmon, Adam Drewnowski, Shenggen Fan, Jessica Fanzo, Line J. Gordon, Frank B. Hu, Liisa Lähteenmäki, Ngozi Nnam, Bradley G. Ridoutt, Juan Rivera, Boyd Swinburn, Pieter van’t Veer

**Affiliations:** ^a^Division of Human Nutrition and Health, Wageningen University and Research, 6700 AA Wageningen, The Netherlands; ^b^Johns Hopkins Center for a Livable Future, Johns Hopkins Bloomberg School of Public Health, Johns Hopkins University, Baltimore, MD 21287; ^c^Montpellier Interdisciplinary Center on Sustainable Agri-Food Sustems, French National Institute for Agriculture, Food, and Environment, International Center for Advanced Mediterranean Agronomic Studies, French Agricultural Research and Cooperation Organization, Montpellier SupAgro, University of Montpellier, 34090 Montpellier, France; ^d^Center for Public Health Nutrition, University of Washington, Seattle, WA 98195; ^e^College of Economics and Management, China Agricultural University, Beijing 100083, China; ^f^Berman Institute of Bioethics, Nitze School of Advanced International Studies, Bloomberg School of Public Health, Johns Hopkins University, Baltimore, MD 21205; ^g^Stockholm Resilience Center, 106 91 Stockholm, Sweden; ^h^Department of Nutrition, Harvard School of Public Health, Boston, MA 02115; ^i^Department of Epidemiology, Harvard School of Public Health, Boston, MA 02115; ^j^Channing Laboratory, Department of Medicine, Brigham and Women’s Hospital and Harvard Medical School, Boston, MA 02115; ^k^Department of Management, Aarhus School of Business and Social Sciences, Aarhus University, 8000 Aarhus, Denmark; ^l^Department of Nutrition & Dietetics, University of Nigeria, 410105 Nsukka, Enugu, Nigeria; ^m^Commonwealth Scientific and Industrial Research Organisation, Agriculture and Food, Clayton South, Canberra ACT 2601, Australia; ^n^Department of Agricultural Economics, University of the Free State, Park West, Bloemfontein 9301, South Africa; ^o^National Institute of Public Health, 62100 Cuernavaca, Mexico; ^p^School of Population Health, The University of Auckland, Auckland 1010, New Zealand

**Keywords:** food system, sustainable diets, perspective, accountability, local vs global

## Abstract

Four years after the EAT-Lancet landmark report, worldwide movements call for action to reorient food systems to healthy diets that respect planetary boundaries. Since dietary habits are inherently local and personal, any shift toward healthy and sustainable diets going against this identity will have an uphill road. Therefore, research should address the tension between the local and global nature of the biophysical (health, environment) and social dimensions (culture, economy). Advancing the food system transformation to healthy, sustainable diets transcends the personal control of engaging consumers. The challenge for science is to scale-up, to become more interdisciplinary, and to engage with policymakers and food system actors. This will provide the evidential basis to shift from the current narrative of price, convenience, and taste to one of health, sustainability, and equity. The breaches of planetary boundaries and the environmental and health costs of the food system can no longer be considered externalities. However, conflicting interests and traditions frustrate effective changes in the human-made food system. Public and private stakeholders must embrace social inclusiveness and include the role and accountability of all food system actors from the microlevel to the macrolevel. To achieve this food transformation, a new “social contract,” led by governments, is needed to redefine the economic and regulatory power balance between consumers and (inter)national food system actors.

Achieving healthy, sustainable, and equitable diets is the defining challenge for 21st-century food systems. Several decades of research have helped to understand the trade-offs between the different dimensions of diet sustainability, and to identify pathways to more sustainable food production processes and more desirable food choices. Recent research has provided a picture of what this planetary diet looks like (1). Consensus and direction, however, are lacking at local and regional levels, as well as on how the concept of environmentally sustainable and healthy diets translates to consumers and their food choices, to policymakers, agriculture, and food industry. In addition, concerns regarding affordability (2) and nutrient adequacies (3) of this recommended pattern have been raised.

The landmark EAT-Lancet report underlined the importance of the dietary shift needed to stay within the various planetary boundaries (1). However, this report did not focus on how to bring about this shift. In addition, to enable the necessary changes to take place, it is essential to understand the conditions for their feasibility at the agricultural, economic, and sociocultural levels. Current disciplinary fragmentation in research is hindering the design and achievement of diets that consider the four domains (health, environment, economy, and sociocultural) in combination, and that consider both food demand and supply. Integrating these domains using systems science could be key to identifying both win-wins and trade-offs in approaches to the diet transition.

This paper aims to discuss perspectives and future directions for achieving healthy and sustainable diets within the sociocultural and geographical subtypes of the food system. Emphasis is on the wider sociocultural and economic systemic drivers influencing food choices, food environments, health, and the environment. Moreover, the added value of a food systems approach and accountability of food system actors is discussed.

## Diets in Context

### The Health and Environmental Setting.

Results from large cohort studies and intervention trials have provided insights linking a variety of dietary exposures (e.g., nutrients, foods, and dietary patterns) with a wide range of health outcomes such as obesity and noncommunicable disease (NCD) risk and premature death. Substantive evidence shows that a high consumption of red and processed meat contributes to higher risk of NCDs and premature death, while they can be substituted with moderate amounts of poultry, seafood, dairy, nuts and seeds, and legumes (4). Moreover, there is strong evidence and biological plausibility to support the beneficial roles of minimally processed plant-based foods including fruits, vegetables, nuts, seeds, whole grains, and legumes (5). Contrarily, ultraprocessed foods and drinks (UPF) which are highly palatable provide high amounts of energy, saturated fat, salt, sugar, and diverse additives while they are easily consumed in large amounts and are associated with obesity and NCDs (6).

The environmental sustainability of diets adds an extra dimension to diet quality. Overall, a hierarchy between food categories and greenhouse gas emissions (GHGE) is observed, with plant-based foods having the lowest and animal-based foods the highest impact per kg of food (7). Incorporating the local context by extending GHGE as a global indicator with locally relevant parameters such as land and water use, eutrophication of waterways, presence of heavy metals or pesticides, and biodiversity losses would add valuable information to better assess cobenefits and trade-offs at local and national levels and develop national food-based dietary guidelines (FBDGs) for sustainable diets (8–10).

### Sustainable Diets Depend on the National Context.

Several studies have highlighted that higher quality of diets (based on nutrients or adherence to dietary guidelines) is not automatically associated with lower GHGE (11–13). For example, low-GHGE diets were often high in sugar and other simple carbohydrates and low in essential micronutrients (13). In many, but not all, high-income countries (HICs), higher scores on diet quality indicators are associated with lower GHGE (10, 14); while in China, for example, it is positively associated (15). Moreover, lower-cost foods such as sugar-sweetened beverages, fried potatoes, and refined grains have relatively low environmental impacts but are also associated with health concerns. To jointly evaluate both health and environmental dimensions in the current food system, Clark et al. (16) examined multiple health outcomes and a composite score of environmental impacts of food categories which summarized the current evidence (Table 1).

**Table 1.** Food categories as related to health and environmental impacts for usual dietary patterns and levels of consumption^*^

Major food categories	Nutritional benefits^†^	Risk of chronic disease & mortality^‡^	Environmental impact^‡^
Plant foods (whole grains, fruits^§^, vegetables^§^, potatoes and tubers^¶^, legumes, nuts, and olive oil)	High	Low	Low
Fish, seafood, and poultry	High	Low	Moderate
Dairy and eggs	High	Neutral to moderate	Moderate
Red and processed meats	Moderate	Moderate to high	High
Sugar-sweetened beverages and refined grains	Low	Moderate to high	Low to moderate

^*^Order in the table based on nutrition, health, and environmental impact, not economy and culture.

^†^based on EFSA nutrient recommendations (17).

^‡^adapted from Clark et al. (18), based on per kilogram estimates.

^§^ranges in environmental impact depend markedly on production method (greenhouse or field grown) and functional unit.

^¶^when boiled or mashed, not if used as fried staple product.

The EAT-Lancet commission presented a planetary health diet as global reference; however, healthy and sustainable diets are culturally diverse and vary depending on individual preferences, household budget, local foods, and cuisine. For instance, modeling studies of diets in France, the Netherlands, and other European countries have shown that there are multiple ways to meet nutritional and environmental requirements by individual food choices (19–22). There is no single solution to which foods and food categories need to be substituted in specific diets, but they share the common features of reducing the animal foods, like beef, which are most damaging to the environment and reducing the UPF and beverages which are most harmful to human health. More nutritious products are often more environmentally sustainable; however, there are exceptions to this relationship (e.g., fish), and foods which consumers may view as substitutable can have very different impacts (23). Context-specific dietary changes therefore depend on the national burden of disease (obesity or undernutrition), environmental challenges, and cultural traditions. For example, increasing meat and dairy consumption would help improve current inadequacies in many low- and middle-income countries (LMICs) while most HICs should limit their consumption because of high NCD risks and environmental footprints.

### Food Properties and Food Choice Context.

Sensory and physiological drivers are among the key motivations behind food choices (24) and play an important role in shifting toward healthy and sustainable diets. Preferences for sensory food properties are learned as part of the cultural socialization process apart from human’s innate preference toward sweetness, fat, umami, and salt. However, these innate preferences are modified by cultural conventions, marketing, and exposure to foods in different social contexts (25). Apart from taste, food texture is crucial to the eating rate (the amount of kcal swallowed/minute), e.g., eating grapes takes more time and is more satiating than the same energy and nutrients from grape juice (26). The current abundance of sweet and energy-dense refined foods and drinks bypasses physiological mechanisms of appetite and satiation and likely contributes to energy imbalance and weight gain (27). Unraveling the interplay between these food properties and dietary patterns might help to understand how food processing could be adapted to contribute to obesity prevention and planetary health.

### Economic and Sociocultural Context.

As incomes rise, the proportion of disposable income spent on food drops (Engels Law, 1857) and the proportion of starchy staples in the diet declines (28). Thus, economic growth along with globalization is leading the nutrition transition and related dietary patterns (29). Low socioeconomic groups have limited financial and physical access to high-quality foods, providing a barrier to preferred food choices (30, 31). Moreover, the trend among LMICs is to replace plant-based proteins (wheat, rice, beans) with animal proteins (red meat, chicken, eggs, and dairy), while there are emerging trends among some HICs in the opposite direction. In Southeast Asia, the traditional diet of fish and rice is now associated with older age, rural settings, and low education and incomes, whereas young urban professionals are more likely to select dairy and chicken (32, 33).

## Diets and Food Systems

### Food System Dimensions.

As evident from definitions by the Food and Agriculture Organisation, sustainable diets are intrinsically linked to sustainable food systems via their relationships to health, environment, culture, and economy (34, 35). In addition, shifts in only production, technology, or dietary changes alone will not be enough for reaching levels within the planetary boundaries (36). It is critical that the scales and actors of the food system be taken into account.

**Fig. 1.**
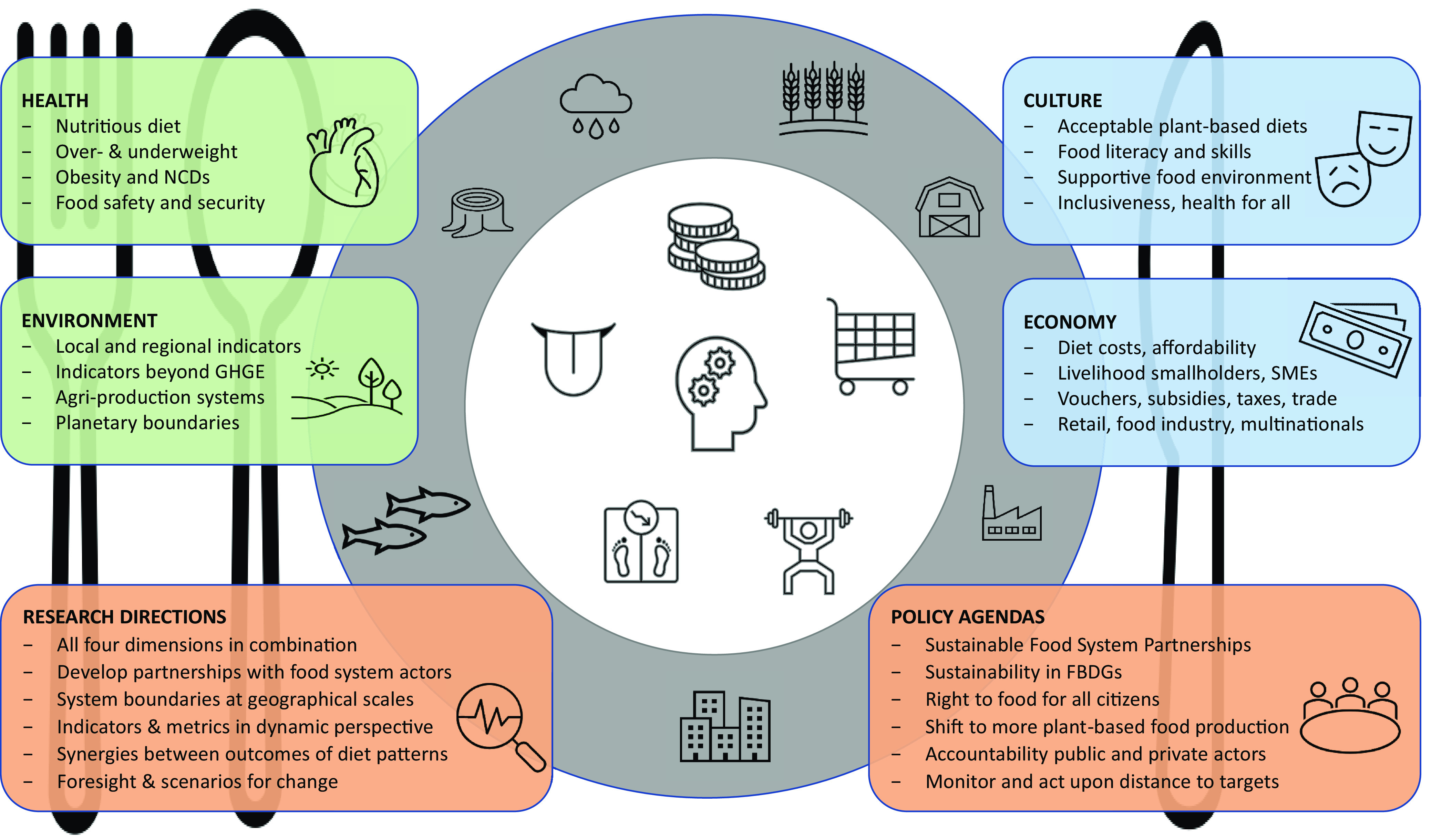
What is on the plate of individuals results from the interplay between the four food system dimensions, scale, and actors. The text boxes list key topics for each of the four dimensions as well as research directions and policy agendas mentioned in this paper (43, 44). Icons on the plate represent factors that directly influence the dietary choices of citizens in their food environment. Icons on the side of the plate represent the natural environment, agriculture, and the built infrastructure as shaped by the behavior of citizens, public policies, and corporate strategies.

Fig. 1 depicts the health and environmental as well as the cultural and economic dimensions as they are related to the food system. The biophysical dimensions set the lower boundaries, while what is actually on the people’s plates is also determined by the built food system. Food shops and retail in the local food environment shape our food choices, dietary habits, and eating culture; food companies and agriculture shape the food supply, largely driven by availability of natural resources and economic principles (34, 35, 37). A sustainable food system supports a healthy life for present and future generations across the globe (35) and is environmentally resilient and economically efficient (38, 39). It is socially inclusive as it ensures access to nutritious foods for the individuals of all incomes, as well as ensuring the livelihoods of smallholders and small and middle enterprises (SMEs) in widely different cultural and economic settings. In line with this, food systems science builds on biophysical requirements for health and planetary boundaries as facts of nature, with cultural traditions and economic laws as modifiable social constructs that support or frustrate “a life on our planet” (40–42).

Moreover, there is no such thing as a single global diet or food system, but there is a multitude of local food systems and individual diets. This is depicted by the inner and outer rings of the plate (Fig. 1). Tackling the global sustainability challenge is as much a societal challenge of food system scales and governance levels as a biophysical challenge of health and environmental boundaries. Thus, system actors and scientists in food, nutrition, and health need to scale-up their focus from “what’s on the plate of the citizens” to the wider food environment that governs food availability, access, and affordability (inner ring). Moreover, they must take into account the wider natural environment, agriculture, and the built infrastructure and the way this is shaped by health concerns versus commercial interests that drive the policy agendas of public and private food system actors.

Third, economic principles govern the behaviors of actors at the different levels of scale of the food system. At the microlevel, individual households buy the foods that are available, accessible, and affordable, contributing to socioeconomic health disparities at the national level and between the Global North and South. At the macrolevel, economic principles match demand of food to regional and global production, but also contribute to environmental problems. The Swedish diet, for example, is largely sourced from international markets, which externalizes the environmental impacts of the Swedish diet across the globe (45). In contrast, in a country like the Netherlands, economically efficient food production reduces net global footprints, but leads to high national impacts of heavy fertilizer use, feed imports, and nitrogen deposition.

### Food System Interactions.

At the microlevel, eating habits originate from the situational interplay between the four dimensions (46). The “natural experiment” of agriculture and socioeconomic development has generated highly culturally divergent solutions to provide nutritious diets while minimizing the risk of acute food shortages but current global trends converge solutions toward a monocultural diet high in UPFs. Crosscultural diversity of dietary patterns (47, 48) offers opportunities to disentangle the infrastructural context and situational heuristics of food choices. Such research can benefit from standardized apps and wearables in transnational studies [65] and will strengthen our understanding of how dietary patterns are determined by consumer characteristics, food environments, and food supply. This will help to identify persistent patterns and their transferability to different food environments.

At the macrolevel of national and regional policies, economic and cultural traditions can be modified to support sustainable diets. Current policies, however, mainly aim to increase consumer awareness and knowledge. Unless combined with a supportive food environment, however, this information-driven approach is clearly not sufficient to change food choices or to drive food producers to change their offerings (49). Small changes by consumers can be aided using labeling or nudging (50), social marketing (51), or apps and wearables (52). Abundant information and good intentions cannot overcome cost constraints caused by the price of higher-quality food items. For instance, production, availability, and affordability of legumes, unrefined cereals, (canned) vegetables, and dairy could go hand in hand with convenient and tasty menu options. As developing such supportive food environments transcends the individual span of control, national policies and regulations need support from taxes, subsidies, or vouchers, adapted to the local context.

### Food System Indicators and Metrics.

Food system research and policies require reliable metrics and indicators to characterize sustainable diets and food systems (53). It has been challenging to develop universally accepted indicators for energy, nutrients, and other dietary components into an overall diet quality score. Examples of food-based scores are the Healthy Eating Index, the Mediterranean Diet Index, Global Diet Quality Score, and the Dietary Approaches to Stop Hypertension index (54, 55). While such scores are consistently associated with lower risk of chronic diseases across different populations (54), advanced diet models would also account for the environmental, economic, and sociocultural dimensions of food systems in globally diverse populations. True cost accounting, for example, could provide market incentives that integrate environmental and health cost, while true pricing and safeguarding purchasing power of consumers could make such diets accessible to all (56, 57).

Environmental indicators, such as land use and water use, are commonly used metrics, but land-use change (e.g., deforestation), water scarcity (58), and biodiversity loss (59) better reflect the short time span in which humanity is approaching the planetary boundaries but less good data is available. Current lifecycle analyses (LCAs) provide attributable footprints and have difficulty incorporating dynamic changes toward circular production systems. Moreover, LCAs often assess the footprints based on current economic value, thereby supporting the status quo of the food system. These issues point at consequential LCAs for use in long-term foresight analyses (60). This method links activities and interdependencies in a product system, and thus allows account for the expected change as a consequence of a change in demand for the functional unit.

Combining the health and environmental dimension raises further issues. Expressing GHGE footprints per kilogram of food, per portion, per 100 kcal, or per protein can dramatically shift the impact order of food categories, but also highlights nutritional issues such as isocaloric, mass-based, or energy density–based interchangeability of protein quantity and quality of foods in the whole diet (61–63). Beyond the food level, indicators of environmental health, acceptability, and affordability are closely intertwined by dietary patterns, which highlights the need of whole-diet approaches.

### Toward Sustainable Diets.

With respect to whole diets, nutrition research has a strong tradition of using mathematical models to optimize dietary patterns, more recently extended to environmental sustainability, both in LMICs (64, 65) and HICs (66–68). These models combine foods into diets, in such a way that they fulfil a desired set of criteria, e.g., health and environment. However, most diet models combine foods as independent entities, whereas in real life, they are consumed in culturally determined meals and snacks; hence, such models have difficulty accounting for the cultural diversity and acceptability of dietary patterns. Other approaches circumvent this problem by considering a priori defined omnivorous, vegetarian, or vegan dietary regimens (69). More recent models addressed food preference, food choice, and cultural acceptability by incorporating costs (70, 71), taste, and texture (72), constraining the deviation from current dietary patterns (73), or making linear combinations of whole diets (68, 74). Approaches that rely on currently existing dietary patterns have the advantage of realism and greater acceptability; conversely, however, they cannot identify radically different solutions that go beyond the current food consumption, composition, and production system.

### Toward Sustainable Supply Chains.

Upstream of the supply chain, further interactions of the food system dimensions must be considered. Food processing (including reformulation and enrichment), packaging, transport, and preparation affect energy and nutrient contents, digestibility, food safety, shelf-life, diet costs, food waste, and health outcomes (75). For example, emerging alternative protein sources can be acceptable to consumers and beneficially affect the LCAs of whole diets, but they do not necessarily improve the health profile. In this domain, private food companies, retail, and SMEs generate unique but usually not publicly accessible data on food ingredients and additives, environmental footprints, trade, and sales. Such data are crucial to understand how the food system simultaneously increases both global food availability and socioeconomic health disparities. Moreover, to link these data to food production and consumption, data harmonization is crucial. Global data on food production, trade, and economics typically refer to food and nutrition security and relate to agricultural commodities and crudely estimated per capita intake of energy and nutrients (76). This contrasts with individual- or household-level food surveys that typically use hundreds of food items to estimate dietary quality of demographic subgroups (77). Understanding the strengths and limitations of these data types and developing diet models linked to agricultural production systems are crucial for a coherent understanding of the interrelated dimensions and scales of the food system (78).

## Actors and Accountability

### Time to Act.

Balancing culture and economy within health and environmental boundaries is critical in order for humans to thrive. Historically, multiple local food systems gradually evolved into the current global system. However, to inclusively and efficiently serve sustainable diets to 9 to 10B people within the Limits to Growth (79, 80), a cultural and economic reset is needed beyond short-term local, national, and regional interests (37). Many food system actors, however, hesitate to make radical system choices and seem to be paralyzed by conflicting interests (governments, food industry) or lack of power and funding [consumers, nongovernmental organizations (NGOs)]. The sustainability mission is mandatory however, and can make a kick-start with 10,000 y of agricultural experience (81). Unless experienced local and powerful global governmental and industry actors take joint responsibility for the transition, the social and economic costs of climate change and poor human health will rise to unmanageable levels.

### Actors: Citizens, NGOs, and Local Communities.

As illustrated by climate strikes and trends toward meat reduction, as well as being aware of hunger, droughts, and humanitarian conflicts impacting food, younger generations are increasingly concerned about the future of our planet. Some citizens take responsibility by engaging in local policies, SMEs, and scale-up local best practices to international food policy networks like the Milan Food Pact (82). NGOs are addressing issues like animal well-being, fair trade, and fossil energy investments. Others become involved in sustainable and organic cooperative farms, whereas advocacy and consumer protection groups have sued national governments for not adhering to global GHGE-targets or European agreements on nitrogen deposition in the environment (83). Thus, whereas (inter)national food system actors hesitate to make necessary system changes, bottom-up initiatives from civil society express dissatisfaction and even distrust in the status quo, aiming to accelerate policy change.

### Actors: Agri-Food Companies and Corporate Strategies.

The green revolution, food technology, and innovation have fostered the transition from informal to formal food markets, contributing to food security of citizens and livelihoods of smallholders, SMEs, and food companies. Nowadays, the food sector provides food and livelihoods to a large number of employees relative to the sales volumes (84). At the same time, smallholders also became dependent on capital-intensive technologies, seed companies, and long-term loans (85). Instead of seasonal food shortages and acute under/malnutrition, local communities now depend on imports, e.g., infant formulae and processed foods, as well as global trade with the accompanying food price spikes and fluctuations (86).

The nutrition transition also shifted the burden of health from acute undernutrition and lack of food safety toward safe foods, excessive energy intake, and chronic micronutrient undernutrition (87). Food companies increased their highly competitive market shares by exploiting sensory preferences for sweet, fat, and salt that span across ethnic, cultural, and geographic boundaries. Their global product portfolio provides humanity with a surfeit of energy-dense and nutrient-poor foods that are cheap compared to whole-food products. For example, in LMICs, the food industry successfully reaches the lowest income consumers via formal and informal distribution channels and marketing strategies, with soft drinks and snacks rather than nutritious foods (31). As high-quality foods are often far more affordable to the rich as compared to the poor, this mainly strikes socioeconomically deprived population groups (2, 37, 88). The resulting health disparities, however, tend to be framed as externalities to the food system and are culturally perceived as responsibilities of individual lifestyle and/or health insurance and governmental income policies.

In response to concerns about sustainability, frontrunners on the food sector are developing innovative plant-based or cultured meat alternatives, while large businesses are essential for the transition by scaling-up production and distribution. Examples are the Vegetarian Butcher (NL), NoMeat (UK), Eat Just (Singapore), and hybrids of plant- and animal-based proteins. The food sector also expresses commitment and sincere ambitions by, for example, procurement of sustainable agricultural commodities and fair-trade strategies. Just as for health claims, however, such initiatives also call for checks and balances from public bodies to prevent greenwashing practices (89, 90).

### Actors: Governments and Public Policies.

For governmental policies, FBDGs are an established way to integrate sustainability into the national culture-specific context, but very few countries have thoroughly done so (91). Mexico was one of the first countries attempting to incorporate the EAT-Lancet principles in its national guidelines (Box 1). The effectiveness of information-driven strategies such as front-of-pack labeling, logos, and education alone, however, is questionable (49, 92). Long-standing knowledge-based efforts to promote healthy eating have shown that this is not an effective strategy, as only a small proportion of any given population adheres to or even knows about the nutritional recommendations (93). Despite laudable intentions, such approaches are based on the erroneous belief that lack of knowledge is the underlying reason for poor food choices. Developing supportive food environments, however, transcends the influence of citizens and is not of primary interest to supply chain actors either. Although public procurement is being explored in schools and canteens, for example, health policies are cautious to interfere in the food environment and focus on food (safety, reformulation, enrichment) rather than the food environment itself.

Box 1. Mexico––translating the EAT–Lancet reference diet to local guidelines

Adapting the generic Eat-Lancet guidelines to the local situation is currently in progress in Mexico. Castellanos et al. have proposed a Mexican healthy and sustainable diet as an adaptation of the global EAT-Lancet reference diet, based on the current dietary patterns of the population. This new reference diet seeks to resemble as much as possible current consumption levels (94), with similar or lower cost (95), alongside local estimates of the environmental impact of the current and healthy and sustainable diets. In addition, they are evaluating the dietary quality and the health impact (Burden of Disease) of these guidelines. The results of this research agenda are being used in the process of updating the Mexican FBDGs to include sustainability as well as sociocultural aspects. Furthermore, in this process, a set of policy actions involving the agriculture, education, welfare, health, and economy sectors were identified, aimed at supporting the adoption of the new guidelines (96). The recommended actions include reinforcing the local production of healthy and sustainable foods and including healthy and sustainable criteria in standards for government procurement of food, shifting current production and consumption subsidies away from unhealthy and nonsustainable foods, improving the implementation and efficiency of a number of regulations and laws aimed at the transformation of food environments (taxes, marketing restrictions, school food standards), and providing information to consumers through warning front of package labels.

In food and agricultural policies, circular production systems can reduce future environmental impact (78). Current policies often prioritize immediate effects of food safety and quality above distant public health and environmental sustainability. Agricultural policies should divert from subsidizing staples, fertilizers, and pesticides to subsidizing sustainable production of commodities that can be processed to an abundancy of healthy foods in affordable diets.

In an economic sense, health and agricultural policies have prioritized competitiveness between supply chain actors and the freedom of consumers to enjoy unsustainable and unhealthy diets. Regulating the supply chain and food environment therefore leads to conflicts between food producers, end users, and civil society. The “invisible hand” in the economy does not guarantee “the wealth of nations” (97) and will require intentionally imposed feedback mechanisms in order to respect population’s health and planetary boundaries. Ultimately, the food systems transition is a global challenge of local limits to growth, where economic interventions such as taxes, subsidies, or vouchers can provide incentives to food system actors to protect the health and environment of current and future generations (98–100).

### Power and Accountability.

Concerns of citizens, policies of governments, and intentions of food companies do not suffice to achieve system change. At each level of the system, the sustainability challenge is hindered by a paralyzing mix of shared and conflicting interests between public and private supply chain actors. Although the adverse impacts of inaction are disastrous, this perpetuates the status quo. The great food systems transformation is not only a scientific challenge but above all a societal challenge for which all food system actors should be held publicly accountable.

At the national level, disparities in health and well-being point at a power imbalance between citizens in local communities, governments, and food companies. Despite their limited power to change the food system, citizens rightfully try to hold public and private actors accountable for economic strategies that can make a difference. It is globally well recognized that corporations have a critical role to play in the transformation (101); however, the current policy settings they work within severely constrain the efforts of companies at the leading edge of change. Failure to install public accountability will aggravate problems (102), and the risk of inaction outweighs scientific uncertainties. This calls for incorporating economic externalities such as health and environmental sustainability into food system reforms. Such measures, however, require a strong political will and economic means of ministries of health, environment, agriculture, and finance to develop intersectoral policies in a wider international framework.

Corporate power is concentrated in a limited number of large supply chain actors (103, 104). Although most food in the global food supply is produced by small- and medium-sizes enterprises locally, only a small number of large seed companies, global commodity traders, and large, transnational food corporations have gained powerful positions and vested interests at decisive points in the food system. (Inter)national governments need to counterbalance this power by trade agreements, taxes and subsidies that incorporate the economic value of health, environmental impacts, and biodiversity (105). Such policies should account for the interrelations between agriculture, equity, justice, food rights, sovereignty, food access, and livelihoods by decent wages so that citizens and smallholders can afford diverse and nutritious diets (106, 107). Because of shared economic interests of food companies and governments, however, current food systems primarily provide foods and livelihoods to those who can afford it while they fall short in fostering long-term health, environment, and inclusiveness (85).

Apart from this deadlock of public and private interest, restoring the balance in the health–environment axis is burdened with tightly anchored governance traditions. For example, the EU subsidiarity principle governs the relation between Member States and the European Commission. To preserve national identity, Member States frame public health as a national responsibility and personal health an individual insurance issue, whereas agri-food production is positioned as a transnational economic interest (108). Fortunately, this issue of governance scales may not frustrate national initiatives. For example, in France, several NGOs, experts, and political organizations are advocating for the implementation of the right to food and the creation of a “social security for food” for the entire population, an innovative system whose originality and strength are to couple the fight against food insecurity and unhealthy eating with economic support to the transition to sustainable food production (109).

Thus, the successful food transition critically depends on societal mechanisms to constrain corporate power where it is acting against the public interests of society in health and the environment. Citizens and private organizations must take responsibility for the global environment and implement habits, policies, and strategies that improve the food system. However, it is the responsibility of governments to lead this transformation in organizations in which accountability across different indicators is arranged and secured. Food system actors should be held accountable for measurable improvements in agriculture, supply chains, justice, food access, and food environments. In this societal transformation, changes in diet will not happen without concomitant adaptation of local food environments, food production, and supply chains. Supply chain actors need to facilitate sustainable choices by a socioeconomic, physical/digital, and technological food environment that supplies affordable and culturally acceptable foods to consumers. Eventually, a healthy and sustainable food system should be considered as a human right and societal responsibility (110, 111).

## Conclusions

Four years after the EAT-Lancet landmark report, it is clear that the food system transition relies on the local implementation of global targets, i.e., into highly specific geographic, social, and economic embedded eating cultures. To this end, food systems research should embrace the local nature of the biophysical (health, environment) and social dimensions (sociocultural, economy) and the accountability of food system actors from the micro to the macro level.

First, local context is key; diets are inherently local and personal. What entails a healthy and environmentally sustainable diet is neither absolute nor universal. Locally diverse climate, geographic, and economic contexts have shaped food production systems and food choices and have become an expression of “learned” social and cultural identity of a given region. The current globalized food system has created a much more homogeneous diet, and any dietary shift going against this identity will have an uphill road. Current research however lacks sufficient insight into taste, texture, satiation, affordability, and perceived health outcomes across national socioeconomic gradients and regional or global food systems. For sustainable diets, affordable foods are not nutritionally or environmentally inferior but they are nutrient dense and have limited footprints. High-quality metrics of what constitutes sustainable and healthy food choices are the foundation to accurately estimate possible trade-offs and implement policies accordingly.

Second, sustainable diets require a systems approach that transcends the consumers’ span of control, call for science to scale-up, minimize fragmentation, and strengthen its interface with stakeholders in food production and food policy. A counter narrative to the current one based on price, convenience, and taste is needed. Diet quality metrics must reflect local realities and values as well as the globalized context of food production, processing, and consumption. In addition, they should incorporate the social value of diets to people, and environmental and health costs can no longer be considered externalities. Public and private stakeholders and global initiatives should embrace social inclusiveness. Trustworthy and independent transnational platforms are required to cocreate effective solutions.

Third, food systems actors are accountable for the transition, in line with their position at points of control in national and international supply chains. Corporate actors have the power, public actors the responsibility, and citizens the right to enjoy ethically responsible and economically affordable foods as a public good. A new “social contract,” initiated by the government, is required to overcome the deadlock by redefining the economic and regulatory power balance between (inter)national food system actors. This should enable public and private stakeholders to scale-up the transition to the systems level, while citizens can create “volume” by buying widely available affordable, healthy, and sustainable foods from supportive food environments.

In conclusion, the global food systems transition calls for local, national, as well as global solutions, based on interdisciplinary research collaboration and transnational scaling-up of national and local policies. This requires a restoration of the balance between economic and public interest, strengthening the regulatory power of public food system actors and increased corporate accountability.

## Data Availability

There are no data underlying this work.
